# Synthesis of Carbon Quantum Dot Nanoparticles Derived from Byproducts in Bio-Refinery Process for Cell Imaging and In Vivo Bioimaging

**DOI:** 10.3390/nano9030387

**Published:** 2019-03-07

**Authors:** Caoxing Huang, Huiling Dong, Yan Su, Yan Wu, Robert Narron, Qiang Yong

**Affiliations:** 1 Jiangsu Co-Innovation Center for Efficient Processing and Utilization of Forest Resources, College of Chemical Engineering, Nanjing Forestry University, Nanjing 210037, China; hcx@njfu.edu.cn (C.H.); suyan1994@njfu.edu.cn (Y.S.); 2 College of Furnishings and Industrial Design, Nanjing Forestry University, Longpan Road 159, Nanjing 210037, China; ll233d@163.com (H.D.); wuyan@njfu.edu.cn (Y.W.); 3 Department of Forest Biomaterials, North Carolina State University, Campus Box 8005, Raleigh, NC 27695-8005, USA; robnarron@gmail.com

**Keywords:** carbon quantum dots, autohydrolyzate, hydrothermal treatment, fluorescence, bioimaging

## Abstract

The carbon quantum dot (CQD), a fluorescent carbon nanoparticle, has attracted considerable interest due to its photoluminescent property and promising applications in cell imaging and bioimaging. In this work, biocompatible, photostable, and sustainably sourced CQDs were synthesized from byproducts derived from a biorefinery process using one-pot hydrothermal treatment. The main components of byproducts were the degradation products (autohydrolyzate) of biomass pretreated by autohydrolysis. The as-synthesized CQDs had a size distribution from 2.0–6.0 nm and had high percentage of sp^2^ and sp^3^ carbon groups. The CQDs showed blue-green fluorescence with a quantum yield of ~13%, and the fluorescence behaviors were found to be stable with strong resistance to photobleaching and temperature change. In addition, it is found that the as-synthesized CQDs could be used for imaging of cells and tumors, which show potential applications in bioimaging and related fields such as phototherapy and imaging.

## 1. Introduction

The carbon quantum dot (CQD), a quantum-sized carbon material, has drawn great attention as a novel material due to its super hydrophilicity, good biocompatibility, and excellent photoluminescence properties [1,2,3,4]. The light-absorbing properity of CQD render them applicable for photocatalysis, energy conversion, optoelectronic devices, and chemical sensors [5,6,7]. In addition, the low toxicity associated with CQD enables them to be harmlessly implemented in instances of cell imaging, in vivo biomaging, and drug delivery systems [4].

Generally, CQD can be manufactured by a “top-down” route from different carbon-based precursors (such as graphite, carbon fibers, and biological sources) [8,9,10]. Strategies for preparing CQD from such materials always involve both chemical oxidation and solvothermal treatment under harsh conditions. Such conditions are necessary in order to oxidize, exfoliate, and diminish the carbon precursors into nanoscale carbonaceous matter. Concentrated acid (such as sulfuric acid and nitric acid) and time-intensive treatment are required to establish the harsh conditions necessary for CQD formulation [11,12,13]. However, these methods are not regarded as green because of the personal safety and environmental hazards associated with the chemistry applied. In an attempt to establish a green and facile process, hydrothermal “bottom-up” methods have attracted growing attention due to this method’s lessened processing severity. The term “bottom-up” refers to synthesis of CQD from molecular precursors such as citric acid, glucose, xylan, or resin [14,15,16]. Hydrothermal synthesis of CQD is a low-cost, facile, rapid, and green method [13]. However, there remains a challenge regarding identifying and sourcing suitable and inexpensive bio-based precursors.

From the precursors investigated across recent literature, it can be inferred that monosaccharides (glucose, xylose, others) from biomass is a renewable and possible precursor to produce the CQD. Lignocellulosic biorefineries can produce monosaccharides by different technologies [17,18]. During a biorefinery process, pretreatment is an essential step required to disrupt the indurative structure of biomass for subsequent enzymatic hydrolysis [19,20]. One pretreatment technology, autohydrolysis, is remarkably green and effective from both a technological and an economic perspective. Autohydrolyis is able to release ~10–20% of glucan and ~50% of xylan from biomass into an aqueous carbohydrate-rich hydrolysate, termed as autohydrolyzate [19]. Because the monosaccharide concentrations in the autohydrolyzate are low and the solution also contains a wide variety of lignin-derived phenolic compounds, autohydrolyzate is often disregarded as a biorefinery waste stream [21]. Hence, we tried to pursue production of CQD from the monosaccharides present in autohydrolyzate. A significant field of research around CQD is their functionalization for bioimaging. One report demonstrated that amino-passivated CQD exhibited strong optical absorption for cell imaging and in vivo biomaging [22]. The application for biomaging was attributed to the enrichment of amine groups at the surfaces of the CQD. Amine-based functionalization agents, such as 1,2-ethylenediamine, trimethylamine, poly(ethylenemine), and butanediamine, have been applied for CQD functionalization across various works [23,24,25]. Urea is a low-cost, low toxicity, and low corrosivity doped agent, which has been used for surface functionalization of bottom-up CQD prepared from sodium citrate and citric acid [26]. However, no work has shown if the various components in the autohydrolyzate can be doped by urea to prepare amino-passivated CQD for biomaging, using the green one-pot hydrothermal treatment as synthesis method. Meanwhile, utilizing the biorefinery byproducts as the precursor to synthesize CQD can potentially show the synthesis process is low cost.

**Scheme 1:**
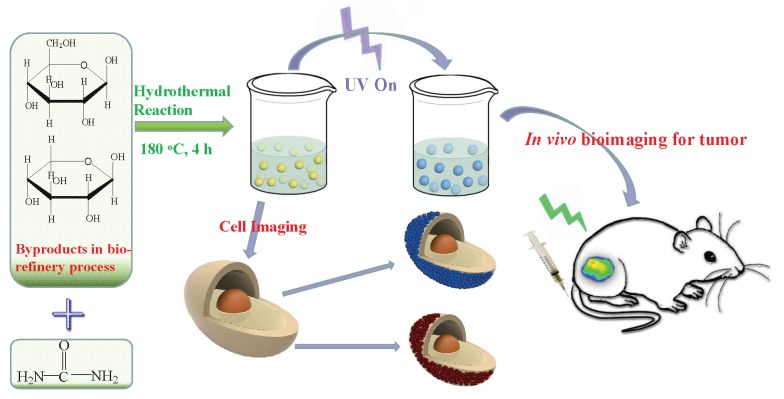
The illustration of the green route for carbon quantum dots (CQDs) synthesis from biorefinery byproducts as the precursor.

In this work, in order to evaluate whether the byproducts in bio-refinery process can be used as the precursors to synthesize CQD, different renewable biomass of wheat straw (WS) and bamboo residues (BR) were used as the raw materials to prepare the autohydrolyzate. The main reactants in the autohydrolyzate are glucose and xylose, which were further mixed with urea to prepare amino-passivated CQDs (CQD-WS (wheat straw) and CQD-BR (bamboo residues)) using one-pot hydrothermal treatment. The optic and physicochemical properties of the as-synthesized CQDs were investigated and their potential applications for cell imaging and in vivo bioimaging were explored, as shown in Scheme 1. The novelty of this work is that the synthesized CQDs from byproducts in bio-refinery process can indeed function as agents for cell imaging and in vivo bioimaging, which have never been reported before.

## 2. Materials and Methods

### 2.1. Materials

Wheat straw and bamboo residues were used as raw materials to prepare autohydrolyzate. The bamboo residues used were provided by the He Qi Cang Bamboo Processing Factory (Fujian, China). Wheat straw was obtained from farm land in Jiangsu province, China.

### 2.2. Synthesis of Carbon Quantum Dot (CQD) Using One-Pot Hydrothermal Treatment

Autohydrolyzates (AH) from wheat straw and bamboo residues were prepared according to our previous work [19], termed AH-WS and AH-BR. Vacuum concentration was applied to the original autohydrolyzate to increase the final concentration of monosaccharides (glucose, xylose, and arabinose) in the AH-WS and AH-BR to ~40 g/L.

One-pot hydrothermal treatment was carried out in a 100 mL bomb heated by a hot oil bath. Inside the bomb, 50 mL of concentrated AH-WS and AH-BR (with 2 g total monosaccharides) was mixed with 6 g urea and heated at 180 °C for 4 h. After time, the bomb was carefully removed from the oil bath and immediately cooled in ice water. After opening the bombs, the liquid and precipitate were separated by centrifugation at 10,000× *g* for 10 min. The liquid was then dialyzed using a dialysis tube (molecular weight cutoff of 8000 g/mol). The material remaining outside of the dialysis tube was denoted as the carbon quantum dots (CQD). The CQD obtained from the autohydrolyzates of AH-WS and AH-BR were termed as CQD-WS and CQD-BR. The CQD-WS and CQD-BR solutions were finally dried by vacuum to produce CQD powders.

### 2.3. Characterization of the Synthesized CQDs

The Fourier transform–infrared (FT–IR) spectra of the CQD samples were recorded on a FT–IR spectrophotometer (VERTEX 80V, Karlsruhe, Germany). Nuclear magnetic resonance (NMR) spectra (^1^H, ^13^C and 2D-HSQC) of CQD samples dissolved in D_2_O were recorded using a Bruker Avance 600 MHz spectrometer (Karlsruhe, Germany). Visual structures of each CQD sample was examined by transmission electron microscopy (TEM) using a JEM 2100 transmission electron microscope (JEOL Ltd., Tokyo, Japan). CQD photoluminescent spectra were recorded using FLS980 fluorescence spectrophotometer (Edinburgh Instruments, Ltd., Livingston, UK) in aqueous solution. The quantum yield of the CQD samples were determined at an excitation wavelength of 360 nm, using quinine sulfate as the reference. X-ray photoelectron spectroscopy (XPS) spectra of CQD samples were also acquired using an Ultima IV spectrometer (Rigaku, Japan).

### 2.4. Cell Viability Evaluation of the Synthesized CQDs

Cell viability of CQD-WS and CQD-BR were estimated using a 3-(4,5-dimethylthiazol-2-yl)-2,5-diphenyltetrazolium bromide (MTT) assay in L929 fibroblasts. In the first step of the assay, fibroblasts were seeded into 96-well plates, with 6000–7000 cells in 200 mL of culture medium per well. The plates were then incubated at 37 °C in a 5% CO_2_ atmosphere for 24 h to allow the cells to attach to the wells. Next, 100 µL of CQD-WS and CQD-BR solution at different concentrations (0, 25, 50, 100, 200 and 400 μg/mL) and 20 µL of MTT solution were added to each well and further incubated at 37 °C for 4 h. After incubation, the absorbance of each well were measured at 490 nm by a microplate reader. All assays were performed at least three times.

### 2.5. Cell Imaging of Synthesized CQDs

To begin the process of cell imaging, murine myeloma cells line SP2/0 were seeded into 6-well culture plates containing 10% calf serum, 2.2 g/L NaHCO_3_, 100 U/mL penicillin, and 100 g/mL streptomycin in 5% CO_2_ at 37 °C for 24 h. Next, 1 mL of the CQD solution at the concentration of 100 μg/mL (dissolved in phosphate buffered saline (PBS), pH = 7.4) was added in the plate and further incubated for 3 h. After incubation, the SP2/0 cells were washed with PBS solution three times to remove extracellular CQD. After washing, the coverslips were placed on a glass microscope slide to affix the cell and cell fluorescence images were acquired using a confocal laser scanning microscope.

### 2.6. In Vivo Bioimaging for Tumor Cell of Synthesized CQDs

The in vivo bioimaging ability of CQD-WS and CQD-BR were examined using mouse experiments conducted according to the regulations of the He Nan Medical Research center. Smmc-7721 tumor cells were used as the tumor that was induced in the nude mouse; 4 nmol of the CQD solution was injected into the nude tumor-bearing mouse by tail vein injection. In vivo fluorescence imaging images (~5 min–24 h) of the nude mouse with tumor and CQD injection were recorded by a NightOwl LB 983 three dimensional small-animal imaging system. After injection with the CQD solution for 24 h, the mouse was euthanized and its major tissue and organs were dissected to obtain additional fluorescence images.

## 3. Results and Discussion

### 3.1. Characterization of As-Synthesized CQDs

**Figure 1:**
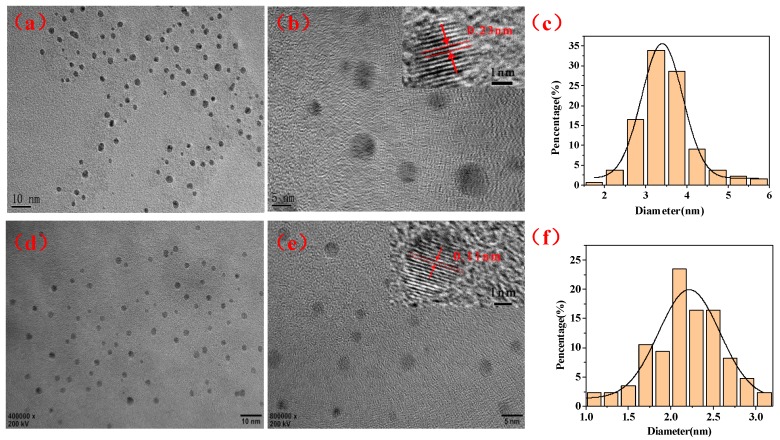
The transmission electron microscope (TEM) (**a**,**d**), high-resolution TEM (HR-TEM) images (**b**,**e**) and particle size distributions (**c**,**f**) of CQD-WS and CQD-BR, respectively.

The green and low-cost precursor byproducts in the bio-refinery process, which are obtained from the autohydrolyzate in a biorefinery waste stream, are reacted in the one-pot hydrothermal treatment for 4 h without adding any strong acids, oxidizers, and metal. Urea is used as doped agent to prepare amino-passivated CQDs from the byproducts. The facile and environmentally friendly treatment resulted in the as-synthesized CQDs possessing a uniform particle size that is less than 8 nm, as shown in the TEM images in Figure 1. Concerning each sample, CQD-WS was revealed to have a narrow distribution mostly in the size range of ~1–6 nm. Their average size was 3.5 nm (Figure 1c). Separately, CQD-BR also comprised a uniform distribution but the particle diameters were smaller ~1–3 nm (Figure 1f). High-resolution TEM shows that most of the CQD-WS and CQD-BR particles contained a well-resolved lattice spacing of 0.23 nm (Figure 1b inset) and 0.11 nm (Figure 1e inset), respectively. From this analysis, it can be concluded that the CQD materials synthesized from the biorefinery pretreatment byproduct are indeed the quantum-sized materials.

**Figure 2:**
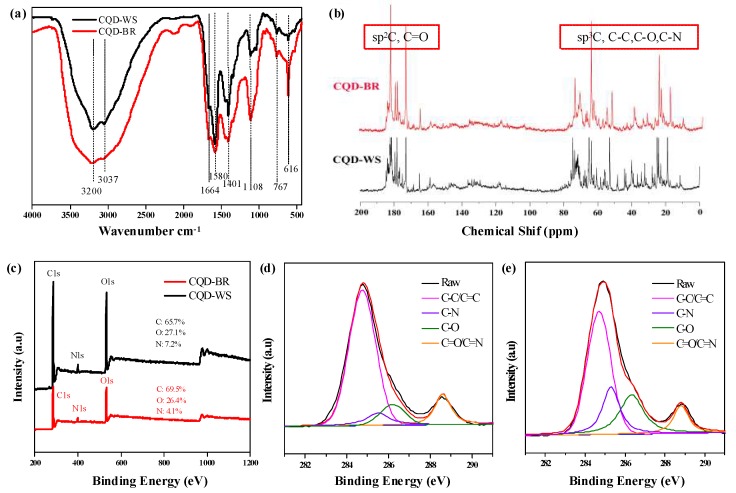
(**a**) Fourier transform–infrared (FT–IR) spectra, (**b**) ^13^C nuclear magnetic resonance (NMR) spectra, (**c**) X-ray photoelectron spectroscopy (XPS) survey spectra of CQD-WS and CQD-BR, (**d**) C 1s XPS spectra of CQD-WS and (**e**) C 1s XPS spectra of CQD-BR.

The functional groups on the surfaces of both CQD samples were investigated by comparing the Fourier transform infrared (FT-IR) spectra obtained and shown in Figure 2a. A broad peak centered at 3200 cm^−1^ corresponds to the stretching vibration and in-plane bending vibration of O-H and N–H. Peaks at 1664 cm^−1^ and 1108 cm^−1^ are attributed to C=O and C–O stretching vibrations, respectively. The absorption band at 1580 cm^−1^ and 1401 cm^−1^ are assigned to the bending vibration of C=C/N–H and C–N stretching. The stretching vibration and bending vibration of C–H were observed at 3037 cm^−1^ and 1108 cm^−1^, respectively. All the information from the FT–IR spectra provides proof that hydroxyl, carboxyl, and amino groups are present at the surfaces of the CQDs [4,13].

NMR spectroscopy (^1^H, ^13^C and 2D-Heteronuclear Single Quantum Coherence (2D-HSQC)) was employed to distinguish the types of hybridized carbon atoms and discernible chemical structures present in the prepared CQDs. In the ^13^C NMR spectra of CQD-WS and CQD-BR (Figure 2b), signals were detected in the range of 15–70 ppm and 100–185 ppm. These signals correspond to both aliphatic carbon atoms (sp^3^) and sp^2^ hybridized carbon atoms, respectively. Signals between 170–185 ppm were attributed to the carbon nuclei of carboxylic acid groups at the surface of the CQD [27]. In the ^1^H NMR spectra (Figure S1), sp^2^ hybridized carbon atoms signals were also found in the range of 7–9 ppm, suggesting both CQD samples possess aromatic structures. This can be also confirmed from their corresponding signals in 2D-HSQC spectra (Figure S2) [28,29], in which the aromatic structures signals can be obviously observed in the region of δ_C_/δ_H_ 100–135/5.5–8.5 ppm.

Surface groups and chemical structures were also investigated by X-ray photoelectron spectroscopy (XPS). The results from XPS spectra (Figure 2c) indicate that both CQD-WS and CQD-BR consist of carbon, nitrogen, and oxygen. This is provided by observation of the following peaks: C 1s, O 1s, and N 1s at 284.9 eV, 532.4 eV, and 399.25 eV, respectively. The C 1s XPS spectra (Figure 2d,e) reveal four peaks at 284.76 eV, 285.4 eV, 286.23 eV, and 288.6 eV for C–C/C=C, C–N, C–O, and C=O/C=N, respectively [4]. All of the analytical results presented thus far indicate that the prepared CQDs possess a naturally conjugated structure with passivated surfaces carrying aliphatic and aromatic hydroxyl groups, carboxylic acid groups and amino groups. These structural features, in particular the surface active amino groups, suggest that the as-prepared CQDs are suitable for luminescence emission.

### 3.2. Photophysical Properties of As-Synthesized CQDs

**Figure 3:**
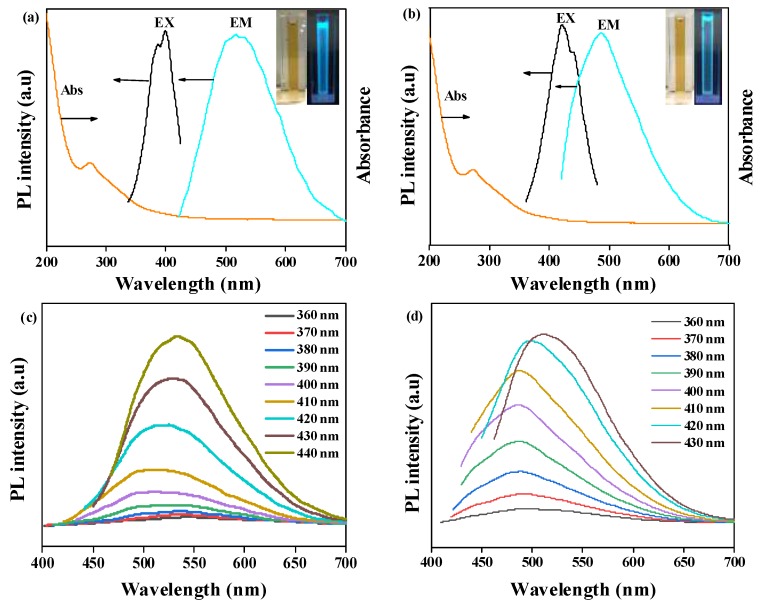
Ultraviolet–visible (UV–Vis), fluorescence excitation and emission spectra of the CQD-WS (**a**) and CQD-BR (**b**); excitation-dependent fluorescence emission of CQD-WS (**c**) and CQD-BR (**d**).

The optical properties of each CQD were characterized to further investigate these material’s suitability for bioimaging. As shown in Figure 3a,b, the ultraviolet–visible (UV–Vis) spectra for both CQD-WS and CQD-BR contain an absorbance peak at 280 nm. This peak is attributed to the π–π* transition of the aromatic carbon bonds. In the Figure 3c,d, it can be seen that the fluorescence intensities of CQD-WS and CQD-BR dependent on the fluorescence emission. Visually, it can be seen that the prepared CQDs are easily dispersible in water and have the appearance of a transparent, uniform, and yellow solution under normal light. However, they exhibit blue-green fluorescence under 365 nm UV light irradiation (Figure 3a,b inset). With quinine sulfate in 0.10 M H_2_SO_4_ as the reference, the synthesized CQDs were measured with quantum yield of ~13% under the excitation of 365 nm, which is a relatively high degree and further proves the material’s viability in bioimaging.

**Figure 4:**
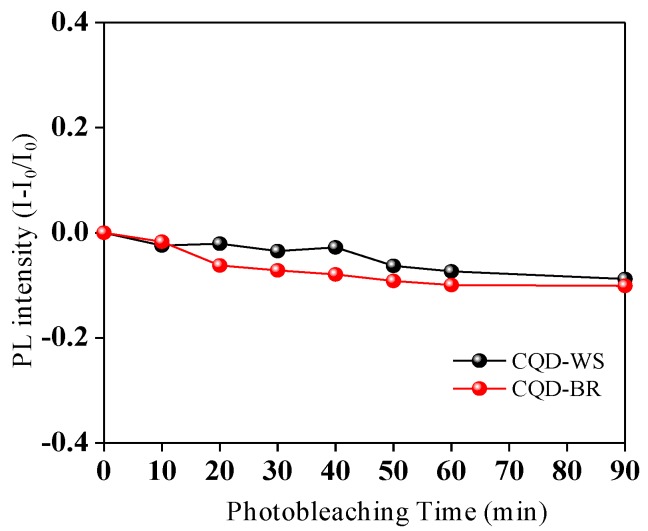
Fluorescence intensity of CQD-WS and CQD-BR upon irradiation with UV light at 365 nm (0.1 mW cm^−2^) in aqueous solution.

The fluorescence emission intensity of the surface state/molecule state of the CQDs is strongly affected by surrounding factors like environmental temperature and UV irradiation. Figure S3 shows the fluorescent behavior of the CQD-WS and CQD-BR observed at 4–60 °C. It can be seen that the temperature showed low effects on the CQD fluorescence properties. Importantly, it is found that there were no obvious changes in fluorescent intensity or peak excursion at different temperatures. When they were irradiated by UV at 365 nm, just a slight decrease in fluorescence intensity (7% and 9%, Figure 4 and Figure S4) occurred after the they were photobleached for 90 min. This decrease was minor compared to 4′,6-diamidino-2-phenylindole (DAPI), a commercial fluorescent display agent currently used in bioimaging. For DAPI, an obvious decrease in fluorescence intensity (about ~45–50%) was found after similar photobleaching treatment [29]. This different indicates that the prepared CQDs are more durable and robust compared to the commercial material. Hence, it is speculated that the CQDs synthesized from a biorefinery liquid byproduct can provide a sustainable alternative chemical in the field of bioimaging.

### 3.3. Biocompatibility Evaluation of As-Synthesized CQDs by Using Fibroblasts and Murine Myeloma Cells

**Figure 5:**
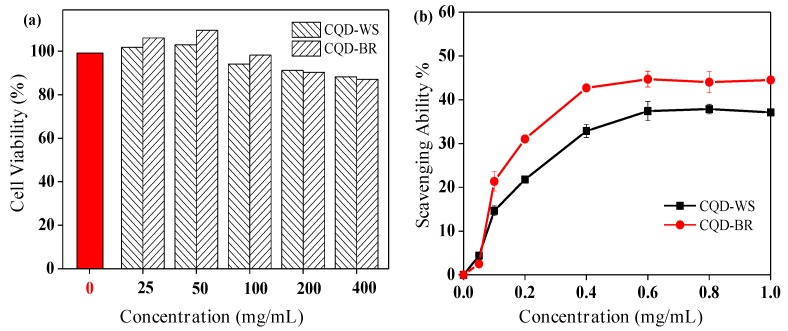
(**a**) Cell viability of L929 fibroblasts after incubation with different concentrations of CQD-WS and CQD-BR for 24 h; (**b**) 2,2-diphenyl-1-picryl-hydrazyl (DPPH) radical scavenging ability of CQD-WS and CQD-BR.

Biocompatibility and bioviability are a crucial factors that must be considered when investigating carbon quantum dot nanoparticles for the bioimaging of a cell [30,31]. To evaluate the biocompatibility behavior of the prepared CQDs, we examined the in vitro cytotoxicity of the samples using an assay involving L929 fibroblasts. As shown in Figure 5a, the cell viability remained greater than 90% when the concentrations of both CQD-WS and CQD-BR increased from 25 to 400 mg/mL. This demonstration indicates that they have excellent biocompatibility, providing further proof that these materials are suitable for bioimaging. The results are in accordance with the biocompatibility of CQDs obtained from different sugars and biomass [4,13]. To explore the biological properties of the CQDs, 2,2-diphenyl-1-picryl-hydrazyl (DPPH) radical scavenging assays were performed to evaluate whether the samples also have any additionally beneficial antioxidant capacity. From Figure 5b, it can be seen that both CQD-WS and CQD-BR exhibited scavenging activities on DPPH radicals in vitro, with the maximum scavenging ability ~40% at 0.6 mg/mL.

**Figure 6:**
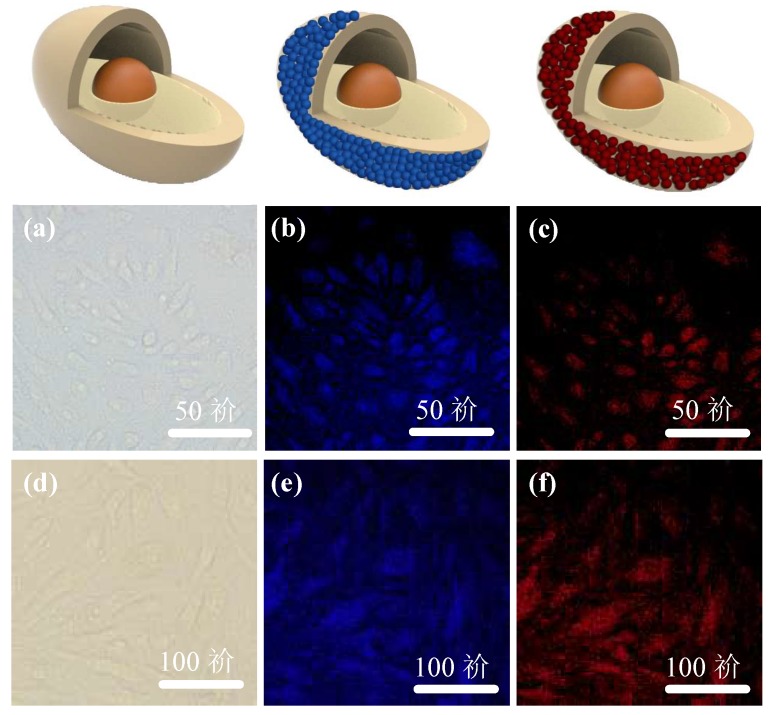
Confocal laser scanning microscopy images of SP2/0 murine myeloma cells at bright-field (**a**,**d**), fluorescence images of the cells incubated with CQD-WS (**b**,**e**) and CQD-BR (**c**,**f**) solution under excitation wavelengths of 420 and 540 nm, respectively.

Based on the good biocompatibility, CQD-WS and CQD-BR were introduced into SP2/0 murine myeloma cells line for in vitro bioimaging by confocal laser scanning microscopy. As shown in Figure 6a,d, the profile of SP2/0 is only slightly observable in the bright-field. However, the cells exhibited the intended blue fluorescence and red fluorescence upon incubation with CQD-WS (Figure 6b,e) and CQD-BR (Figure 6c,f) at excitation wavelengths of 420 and 540 nm, respectively. These results indicated that the synthesized CQDs from byproducts in the bio-refinery process can indeed function as bioimaging agents.

### 3.4. Bioimaging Applications for Tumor Cell of As-Synthesized CQDs

**Figure 7:**
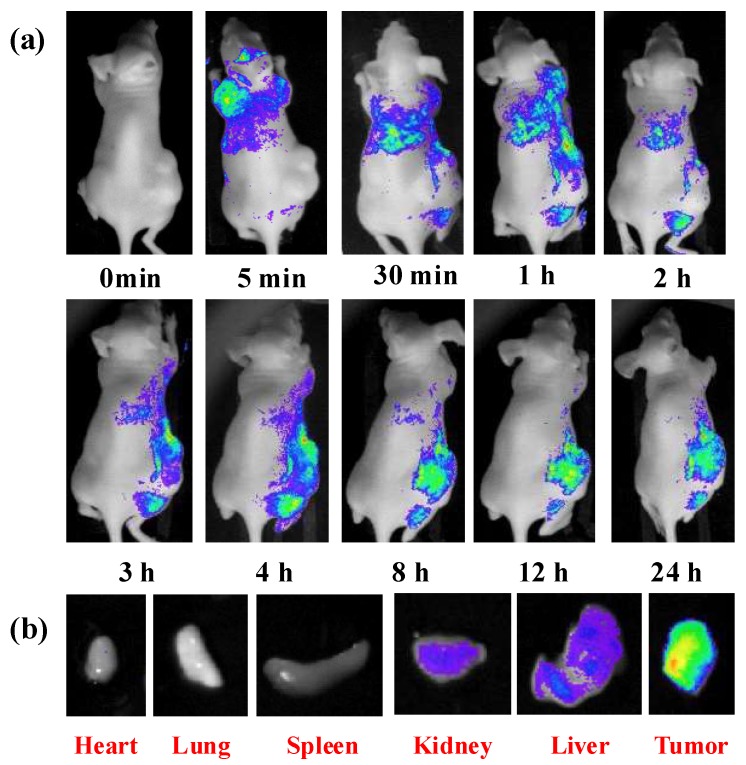
(**a**) In vivo fluorescence imaging of nude mice after intravenous injection of CQD-WS solution; (**b**) representative fluorescence images of dissected organs of a mouse after intravenous injection of CQD-WS solution for 24 h.

For the in vivo optical bioimaging studies, a nude mouse was inoculated with Smmc-7721 tumor cells. The optical imaging of the CQD-WS was investigated by intravenous injection of 200 uL CQD-WS (0.2ug/mL) into the mouse via the tail vein. Optical images of distribution of the CQD-WS in the tumor-bearing mouse over increasing times are shown in Figure 7a. In addition, optical images of fluorescence intensities within the harvested organs are shown in Figure 7b. Without the injection of CQD-WS, no fluorescent signals were detected in the tumor-bearing mouse’s body (Figure 7a, 0 min). 5 min after injecting the CQD-WS solution, fluorescence signals gradually became detectable, first at the head of the mouse. As time progressed towards 3 h, the CQD-WS circulated in the mouse’s body and gradually congregated at the location of the tumor. This observation indicates that the CQD-WS can cycle in a mouse’s body and also aggregate where it is intended to. After 12 h, it was found that CQD-WS stabilized almost exclusively at the location of the tumor. This is another demonstration that the CQD has good biocompatibility and could be used for tumor bioimaging over prolonged periods of time. Figure 7b shows that the dissected kidney, liver, and tumor from the euthanized mouse showed fluorescence, revealing that the CQD-WS was taken up by these organs [32,33]. However, no fluorescence signals were detected in the organs of the heart, lung, and spleen. Overall, most of the CQD-WS aggregated in the tumor and fluoresced intensely, indicating that the as-synthesized CQD-WS was a viable bioimaging agent and thus has immense potential in related fields such as phototherapy and bioimaging.

## 4. Conclusions

CQDs with narrow size distribution from 2–6 nm were successfully synthesized using a one-pot hydrothermal reaction using a biorefinery byproduct (autohydrolyzate) doped with urea. NMR, FT–IR, and XPS spectroscopy confirmed the associated chemical moieties in the CQDs had high percentages of sp^2^ and sp^3^ groups. The CQDs showed stable fluorescence behavior, even under changing temperatures and undergoing photobleaching treatment. In addition, in vivo cell imaging and biomaging experiments showed that the CQDs have low cytotoxicity, antioxidant activity, and an overall excellent bioimaging performance. In all, our work demonstrates a novel means of producing high-value materials that can be applied in advanced bioimaging applications using only a liquid byproduct from biorefinrey processes.

## Supplementary Materials

The following are available online at https://www.mdpi.com/2079-4991/9/3/387/s1, Figure S1: ^1^H NMR spectra of CQD-WS and CQD-BR, Figure S2: 2D-HSQC spectra of CQD-WS and CQD-BR, Figure S3: the fluorescent behavior of the CQD-WS and CQD-BR observed at 4–60 °C, Figure S4: Fluorescence intensity of CQD-WS and CQD-BR upon irradiation with UV light at 365 nm (0.1 mW cm^−2^) in aqueous solution.
