# Supplementary material for: Synthesis of Carbon Quantum Dot Nanoparticles Derived from Byproducts in Bio-Refinery Process for Cell Imaging and In Vivo Bioimaging
Source: Nanomaterials (Basel). 2019 Mar 7;9(3):387. doi: 10.3390/nano9030387 (PMC6473984; doi:10.3390/nano9030387)
Supplement: Supplementary file 1 [file nanomaterials-09-00387-s001.pdf]

Synthesis of carbon quantum dot nanoparticles derived from byproducts in bio-refinery process for cell imaging and in-vivo bioimaging

Caoxing Huang [1#], Huiling Dong [2#], Yan Su [1], Yan Wu[2], Robert Narron[3], and Qiang Yong [1,*]

[1] Co-Innovation Center for Efficient Processing and Utilization of Forest Products, College of Chemical Engineering, Nanjing Forestry University, Nanjing 210037, hcx@njfu.edu.cn (C.H.); suyan1994@njfu.edu.cn (Y.S.);

[2] College of Furnishings and Industrial Design, Nanjing Forestry University, Longpan Road 159, Nanjing 210037, China, ll233d@163.com (H.D.); wuyan@njfu.edu.cn (Y.W.);

[3] Department of Forest Biomaterials, North Carolina State University, Campus Box 8005, Raleigh, NC 27695-8005 USA, robnarron@gmail.com (R.N.)

* Correspondence: swhx@njfu.com.cn (Q.Y.); Tel.: +86 25 85427797

[#] Huiling Dong contributed equally to this work, regarding as the first author.

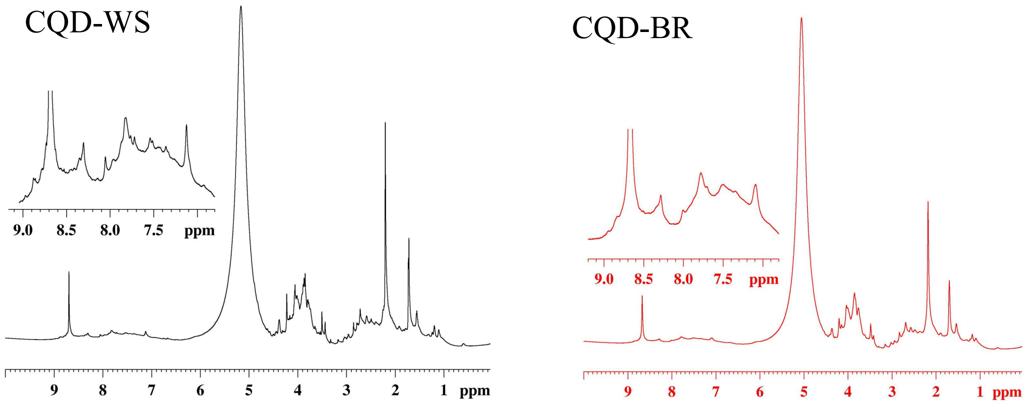

Fig. S1 ^{1}H NMR spectra of CQD-WS and CQD-BR

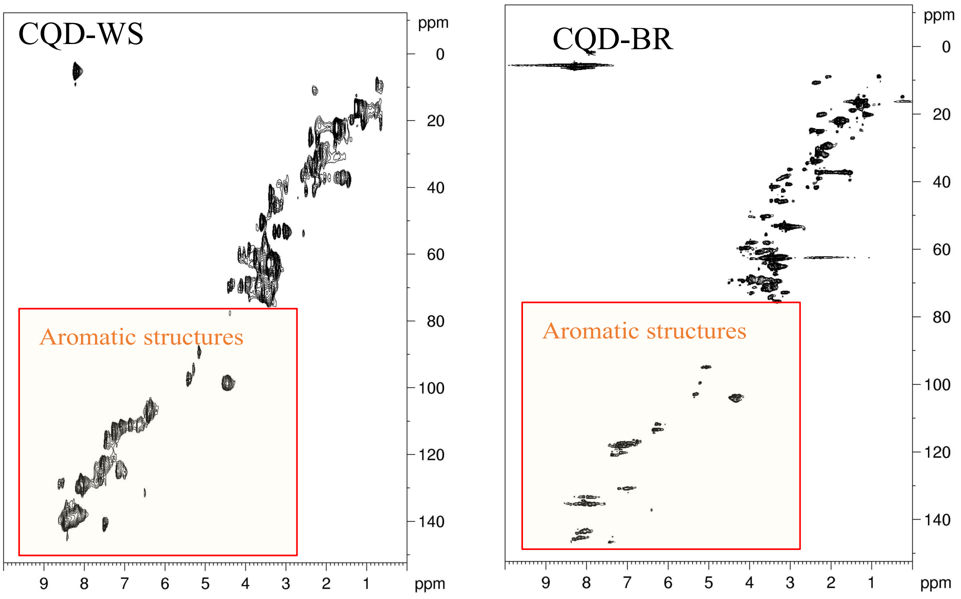

Fig. S2 2D-HSQC spectra of CQD-WS and CQD-BR

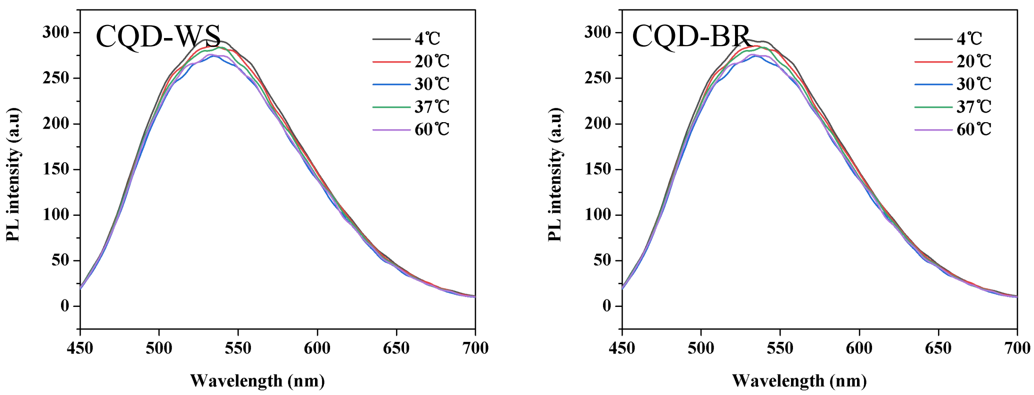

Fig. S3 the fluorescent behavior of the CQD-WS and CQD-BR observed at 4-60 ^{o}C

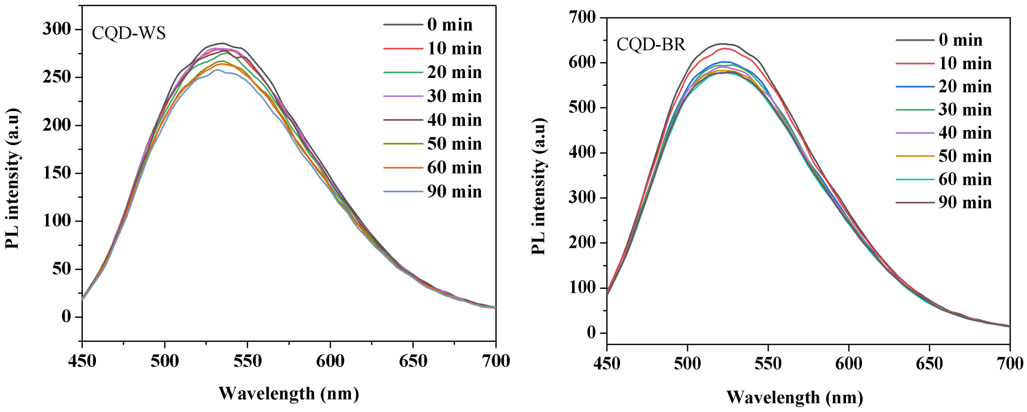

Fig. S4 Fluorescence intensity of CQD-WS and CQD-BR upon irradiation with UV light at 365 nm (0.1 mW cm^{-2}) in aqueous solution
